# Distribution of killer cell immunoglobulin-like receptors genes in the Italian Caucasian population

**DOI:** 10.1186/1479-5876-4-44

**Published:** 2006-10-27

**Authors:** A Bontadini, M Testi, MC Cuccia, M Martinetti, C Carcassi, A Chiesa, E Cosentini, E Dametto, S Frison, AM Iannone, C Lombardo, A Malagoli, M Mariani, L Mariotti, L Mascaretti, L Mele, V Miotti, S Nesci, G Ozzella, D Piancatelli, G Romeo, C Tagliaferri, S Vatta, M Andreani, R Conte

**Affiliations:** 1 Transfusion Service, S. Orsola-Malpighi Hospital, Bologna, Italy; 2 Mediterranean Institute of Hematology, Rome, Italy; 3 Italian KIR Collaborative AIBT Group, Italy

## Abstract

**Background:**

Killer cell immunoglobulin-like receptors (KIRs) are a family of inhibitory and activatory receptors that are expressed by most natural killer (NK) cells. The KIR gene family is polymorphic: genomic diversity is achieved through differences in gene content and allelic polymorphism. The number of KIR loci has been reported to vary among individuals, resulting in different KIR haplotypes. In this study we report the genotypic structure of KIRs in 217 unrelated healthy Italian individuals from 22 immunogenetics laboratories, located in the northern, central and southern regions of Italy.

**Methods:**

Two hundred and seventeen DNA samples were studied by a low resolution PCR-SSP kit designed to identify all KIR genes.

**Results:**

All 17 KIR genes were observed in the population with different frequencies than other Caucasian and non-Caucasian populations; framework genes *KIR3DL3*, *KIR3DP1*, *KIR2DL4 *and *KIR3DL2 *were present in all individuals. Sixty-five different profiles were found in this Italian population study. Haplotype A remains the most prevalent and genotype 1, with a frequency of 28.5%, is the most commonly observed in the Italian population.

**Conclusion:**

The Italian Caucasian population shows polymorphism of the KIR gene family like other Caucasian and non-Caucasian populations. Although 64 genotypes have been observed, genotype 1 remains the most frequent as already observed in other populations. Such knowledge of the KIR gene distribution in populations is very useful in the study of associations with diseases and in selection of donors for haploidentical bone marrow transplantation.

## Background

Killer cell immunoglobulin-like receptors (KIRs) are glycoproteins expressed on the cell surface of natural killer (NK) and subsets of T cells. These polymorphic receptors interact with specific motifs on HLA class I molecules, modulate NK cytolytic activity and are encoded by genes located on chromosome 19q13.4 [1-3].

They have been divided into distinct groups, depending on the number of external immunoglobulin domains (2D or 3D). The presence of a long cytoplasmatic tail with two immune tyrosine-based inhibitory motifs (ITIMs) allows the transduction of inhibitory signals and characterizes the inhibitory KIRs (2DL, 3DL), whereas the presence of short cytoplasmatic tails corresponds to the activating KIR receptors (2DS, 3DS) [1-4].

Due to the functional link between HLA class I molecules, KIRs are expected to display a high polymorphism. Studies on KIR genes in different populations, along with HLA system evaluations, can provide valuable information about the history and geography of human genes, while a wide range of genotypes may be expected in the human populations [5-8].

Most KIR haplotypes belong to one of two broad groups, termed A and B. The haplotypes have a framework of three conserved blocks containing *KIR3DL3*, *KIR2DL4 *and *KIR3DL2 *and differ in the number and type of KIR genes. Group A haplotype has a unique combination of seven KIR genes, while B haplotype exhibits a substantial variability in KIR gene number. Both groups of genotypes have been found in all populations analysed so far, but their distribution varies considerably among ethnic groups [5,9,10].

Several studies have associated KIR genes with disease susceptibility, immune responsiveness and events following allogenic transplantation; recent reports have implicated KIRs in affecting the outcome of hematopoietic stem-cell transplantation [11].

The aim of this multicentric study was to determine the percentage of the population positive for individual KIR genes and the genotype pattern in a representative ethnic Italian group of 217 samples, using a new low-resolution, gene-level KIR PCR-SSP typing; we compare the data so obtained with those previously described in other Caucasian and non-Caucasian populations

## Materials and methods

217 unrelated healthy Italian individuals from 22 immunogenetics laboratories located in northern, central and southern regions of Italy were randomly selected to represent a fair, representative ethnic Italian group.

Each laboratory typed 10 DNA samples. DNA was previously extracted from peripheral blood samples drawn in EDTA anticoagulant tubes by standard protocols (e.g. salting-out); the DNA concentration and the ratio were evaluated. All participating laboratories performed KIR typing using the same low resolution PCR-SSP assay and the same lot number (KIR Genotyping Kit, Dynal Biotech, Pel-Freez Clinical Systems, Brown Deer, WI, USA) following the manufacturer's instructions. The kit was designed to identify 14 KIR genes (*2DL1, 2DL2, 3DL1, 2DL4, 2DL5, 2DS1, 2DS2, 2DS3, 2DS4, 2DS5, 3DL1, 3DL2, 3DL3, 3DS1*), 2 pseudogenes (2DP1 and 3DP1) and the common variants of *KIR2DL5 *(*KIR2DL5A*, *KIR2DL5B*), the *KIR2DS4 *allele (*001/002 and *003) and *KIR3DP1 *allele (*001/002 and *003).

The specificity of the assay was previously tested by all participating laboratories on a panel of two shared DNA chosen by two reference laboratories to cover the amplification of all mixes included in the typing kit.

PCR-SSP was performed following the manufacturer's instructions. Briefly, 25 μl of DNA at a concentration of 75–125 ng/μl with ratio > 1.5 was mixed with 150 μl of PCR Buffer, 2.4 μl of Taq polymerase and 85 μl of distilled water. Eight μl of this mixture was added to each mix except for the contamination control where a mixture without DNA was used.

Genomic polymerase chain reaction analysis was performed under the conditions recommended by the manufacturers: 1 minute at 95°C for one cycle followed by 30 cycles formed of 20 seconds at 94°C, 20 seconds at 63°C and 90 seconds at 72°C. At the end a constant temperature of 4°C was maintained.

PCR products was transferred to the well of a 2% agarose gel capable of resolving 50–2000 base pair fragments of DNA. The DNA separation was performed at 150 volts for 22–25 minutes. The amplification was checked on a UV transilluminator and photographed. The typing was interpreted by a worksheet.

Statistical analysis was performed as follows: the percentage of the population positive for KIR genes was determined by direct counting. Differences between populations were compared by *χ*^2 ^and Yates-corrected *P- *values for the number of loci investigated.

Linkage disequilibrium (Δ) for two loci associations was calculated according to Mattiuz et al. [12]. The relative linkage disequilibrium (Δ_r_), which gives an indication of the strength of associations between pairs of sequences, was calculated for each pair using Δ_max _and the observed two-locus haplotype frequency [13]. The differences between the observed and expected haplotype frequencies were compared by Yates *χ*^2 ^contingency tables.

## Results

All participating laboratories had the same typing of the two shared DNA. No ambiguities were observed and no false or negative results were described by the laboratories.

The frequencies of the KIR genes are compared to other Caucasian and non-Caucasian populations in table 1.

**Table 1:** Comparison of the KIR gene frequency observed in the Italian multicentric study with Caucasian and non-Caucasian populations.

*KIR *gene	Italian Study (n.217) (%)	Australian Caucasian Witt et al. (n.145) (%)	Irish Crum et al. (n.90) (%)	Germany Uhrber et al. (n.120) (%)	German Becher et al. (n.90) (%)	Greek Niokou et al. (n.233) (%)	North Indian Rajalingam et al. (n.72) (%)	Vietnamese Toneva et al. (n.59) (%)	Australian Aborigine Toneva et al. (n.67) (%)	Thailandia Norman et al. (n.119) (%)	Palestinian Norman et al. (n.105) (%)	Afro Caribbean Cook et al. (n.54) (%)	Asian Cook et al. (n.35) (%)	Chinese Han Jiang et al. (n.104) (%)	Japanese Yawata et al. (n.41) (%)
*2DL1*	95	96	§84 *P0.009*	93	87	89	91	98	§72 *P0.0001*	97	§83 *P0.004*	90	86	99	100
*2DL2*	53	§31 *P0.0001*	50	56	50	50	79 *P0.0001*	37	78 *P0.003*	42	62	54	54	17 *P0.0001*	§17 *P0.0001*
*2DL3*	88	95	90	87	§65 *P0.0001*	88	91	98	§65* P0.0001*	97	85	91	86	99 *P0.0001*	100
*2DL5A*	33	nt	nt	#44	#79	nt	nt	nt	nt	nt	nt	#33	#26	31	#39
*2DL5B*	28	nt	Nt	nt	nt	nt	nt	nt	nt	nt	nt	nt	nt	6 *P0.0001*	nt
2DS1	36	51	56	37	39	43	54	38	82 *P0.0001*	42	44	17	49	34	34
*2DS2*	53	51	51	62	49	54	62	41	84 *P0.0001*	44	64	52	57	17 *P0.0001*	§17 *P0.0001*
*2DS3*	33	28	27	28	22	37	43	31	81 *P0.0001*	25	37	24	29	12 *P0.0001*	§17 *P0.0001*
2DS4 *001-*002	33	#nt	#nt	#nt	#nt	#nt	#nt	#nt	#nt	#nt	#nt	#nt	#nt	80 *P0.0001*	#nt
2DS4* 003	89	92	96	98	96	88	80	87	81	87	88	78	83	42 *P0.0001*	96 *P0.0001*
*2DS5*	28	nt	35	26	27	21	47 *P0.002*	nt	nt	23	27	30	51 *P0.008*	23	28
*3DL1*	96	96	92	96	§87 *P0.0003*	90	91	89	§57 *P0.0001*	93	88	98	91	94	97
*3DS1*	35	nt	Nt	nt	36	46	39	41	78 *P0.0001*	44	39	17	40	32	33

§ With the exception of the Chinese Han population, the primers used were not the same as in the Italian study and hence cannot afford a real comparison. However, any higher percentages of detection are significant. # It should be noted that values given for 2DL5 and 2DS4*003 may contain 2DL5B and 2DS4*001/002 positivities where the latter alleles are reported as not-tested (nt).

The *KIR2DL4*, *KIR3DL2, KIR3DL3 *framework genes were found in all individuals whereas all other inhibitory and non-inhibitory genes were present in a varying percentage of individuals.

*KIR2DL1 *gene was present in 95% of the Italian Caucasian population, while in the Australian Aborigine, Palestinian and in Irish populations it is found at the lowest frequency (72, 83 and 84 percent, respectively) [8,14,15]. The frequency of *KIR2DL2 *was 53% in the Italian population which was higher than in the Australian Caucasian, Chinese Han and Japanese but lower than the Australian Aborigine (78%) and North Indian (79%) populations [6,8,16-18]. *KIR2DL3 *and *KIR3DL1 *have a frequency similar to that observed in the Caucasian population, except for a German regional population described by Becker and in the Aborigine population which were both lower [8,19,20]; in Chinese Han and Japanese populations the frequency was higher [17,18].

The non-inhibitory *KIR2DS1, KIR2DS2, KIR2DS3 *genes have a frequency lower than observed in the Australian Aborigine population (p < 0.005) but higher than in the Chinese Han and Japanese population, though only for *KIR2DS2 *and *KIR2DS3 *[8,17,18].

*KIR2DS4 *gene was present in a high percentage in the Italian population; the expression of allele *003 which identifies the gene variant was present in 89% of subjects studied, as compared to alleles *001 and *002, which are only represented in 33% of the population [21]. A reverse positivity of the alleles was observed to that of the Chinese Han population [17].

*KIR2DS5 *gene has a frequency similar to other Caucasian populations, but is lower than that observed in Asian populations, while *KIR3DS1 *has a frequency similar to other Caucasian and non-Caucasian populations except in the Aborigines in whom it is present in a double percentage [7,8,16].

Linkage distribution (LD) and relative LD values show significant differences between individual KIR pairs (Table 2).

**Table 2:** Linkage disequilibrium values of KIR associations.

		2DL2	2DL3	2DL5A	2DL5B	2DS1	2DS2	2DS3	2DS4* 001-002	2DS4* 003	2DS5	3DL1	3DS1
2	Δ	-0.004	0.097	0.025	0.019	0.01	0.004	0.036	0.03	0.001	0.006	0.031	0.003
D	R	-0.055	0.69	0.597	0.586	0.241	0.055	0.924	0.761	0.011	0.193	0.201	0.064
L	H	0.246	0.623	0.168	0.14	0.15	0.246	0.18	0.117	0.539	0.115	0.685	0.159
1	P		< 0.0001					< 0.05					

2	Δ		-0.137	0.014	0.101	0.013	0.205	0.099	0.012	-0.031	0.003	-0.005	0.025
D	R		-1.186	0.106	0.990	0.096	0.944	0.811	0.1	0.295	0.026	0.087	0.196
L	H		0.071	0.07	0.149	0.077	0.304	0.156	0.071	0.181	0.05	0.252	0.087
2	P		< 0.0001		< 0.0001		< 0.0001	< 0.0001					

2	Δ			0.001	-0.096	0.001	-0.115	-0.086	-0.022	0.025	-0.016	0.007	-0.028
D	R			0.022	-1.774	0.012	-1	-1.333	-0.347	0.120	-0.301	0.054	-0.407
L	H			0.12	0.005	0.135	0.092	0.032	0.1	0.471	0.084	0.549	0.102
3	P				< 0.0001		< 0.001	< 0.001					

2	Δ				0.02	0.135	0.022	0.04	-0.05	-0.052	0.108	-0.051	0.133
D	R				0.162	0.886	0.167	0.269	-0.341	-0.835	0.887	-1.35	0.863
L	H				0.047	0.171	0.078	0.071	-0.016	0.069	0.135	0.096	0.168
5A	P					< 0.0001		< 0.001	< 0.0001	< 0.05			

2	Δ					0.019	0.104	0.119	-0.01	-0.022	0.01	-0.035	0.018
D	R					0.162	1.022	0.969	-0.078	-0.445	0.071	-1.157	0.147
L	H					0.050.	0.152	0.146	0.02	0.08	0.032	0.1	0.048
5B	P						< 0.0001	< 0.0001					

2	Δ						0.021	0.028	-0.05	-0.08	0.115	-0.047	0.127
D	R						0.156	0.195	-0.348	-1.212	0.962	-1.18	0.839
S	H						0.085	0.064	0.012	0.056	0.146	0.119	0.167
1	P							< 0.05	< 0.0001	< 0.05	< 0.0001		< 0.0001

2	Δ							0.106	0.016	-0.02	0.01	-0.005	0.025
D	R							0.868	0.132	0.185	0.103	-0.087	0.196
S	H							0.163					
2	P							< 0.0001	0.075	0.192	0.058	0.252	0.087

2	Δ								-0.011	-0.032	0.003	-0.051	0.04
D	R								-0.076	-0.537	0.028	-1.425	0.275
S	H								0.022	0.09	0.031	0.096	0.075
3	P												< 0.001

2	Δ									-0.107	-0.039	0.0003	-0.039
D	R									-1.811	-0.321	0.009	-0.271
S	H									0.019	0.011	0.155	-0.003
4*001 *002	P									< 0.0001	< 0.01		< 0.05

2	Δ										-0.061	0.108	-0.07
D	R										-1.24	0.804	-1.108
S	H										0.041	0.661	0.063
4*003	P										< 0.05	< 0.000	< 0.05

2	Δ											-0.056	0.105
D	R											-1.876	0.862
S	H											0.068	0.135
5	P											< 0.05	< 0.0001

3	Δ												-0.076
D	R												-2.01
L	H												0.085
1	P												< 0.05

Δ = linkage disequilibrium parameters;R = relative linkage disequilibrium;H = estimated two locus haplotype frequency;P = p value

Among the main inhibitory KIRs, we showed a positive linkage disequilibrium between *KIR2DL1 *and *KIR2DL3 *whereas *KIR2DL2 *is in negative linkage with *KIR2DL3*. About the only non-inhibitory gene observed in genotype 1, KIR2DS4*001/002 is negatively linked with its variant KIR2DS4*003.

The main pairs of KIR genes that show a significant positive and negative linkage disequilibrium are summarized in table 3.

**Table 3:** Pairs of KIR genes that show significant positive and negative linkage disequilibrium

Positive linkage disequilibrium	Negative linkage disequilibrium
2DL1-2DL3, 2DL1-2DS3	2DL2-2DL3
2DL2-2DL5B, 2DL2-2DS2, 2DL2-2DS3	2DL3-2DL5A, 2DL3-2DS2, 2DL2-2DS30
2DL5A-2DS1, 2DL5A-2DS3	2DL5A-2DS4*001-02, 2DL5A-2DS4*003
2DL5B-2DS2, 2DL5B-2DS3	2DS1-2DS4*001-002, 2DS1-2DS4*003
2DS1-2DS3, 2DS1-2DS5, 2DS1-3DS1	2DS4*001-002-2DS4*003,
2DS2-2DS3	2DS4*001-002-2DS5, 2DS4*001-002-3DS1
2DS3-3DS1	2DS4*003-2DS5
2DS4*003-3DL1, 2DS4*003-3DS1	2DS5-3DL1
2DS5-3DS1	3DL1-3DS1

The KIR repertoire is substantially influenced by the polymorphic and polygenic nature of the KIR gene. In all, 65 different profiles were found in the Italian population without pseudogenes and 74 if we include *KIR2DP1 *and *KIR3DP1 *among the 217 samples tested.

Genotypes containing between 6 and 15 genes are included. Genotypes with seven and eleven genes are the most represented in the study population, respectively in 44 and 45 cases.

Following the Uhrberg classification, out of the 65 genotypes identified the most frequent are genotypes 1, 2, 3, 4, 6, 7, 12, 5 and 9 with a frequency of 28.5%, 12.9%, 9.2%, 8.3%, 4.6%, 3.6%, 3.6%, 2.3% and 1.3% respectively (Table 4) [4].

**Table 4:** Distribution of the main phenotypes observed in the Italian population. The nomenclature system from Uhrberg is used for describing the patterns (5). Each genotype is also shown in the different patterns due to the distribution of *KIR2DS4 *alleles and the *KIR2DL5 *variants.

Genotypes	Number	Frequency %	2	2	2	2	2	2	2	2	2	2	2	2	3	3	3	3
			D	D	D	D	D	D	D	D	D	D	D	D	D	D	D	D
			L	L	L	L	L	L	S	S	S	S	S	S	L	L	L	S
			1	2	3	4	5	5	1	2	3	4	4	5	1	2	3	1
							A	B				*001–*002	*003					
1	39	28.5	+	-	+	+	-	-	-	-	-	-	+	-	+	+	+	-
1	20		+	-	+	+	-	-	-	-	-	+	+	-	+	+	+	-
1	3		+	-	+	+	-	-	-	-	-	+	-	-	+	+	+	-
2	11		+	+	+	+	-	-	-	+	-	-	+	-	+	+	+	-
2	16	12.9	+	+	+	+	-	-	-	+	-	+	+	-	+	+	+	-
2	1		+	+	+	+	-	-	-	+	-	+	-	-	+	+	+	-
3	16	9.2	+	+	+	+	-	+	-	+	+	-	+	-	+	+	+	-
3	4		+	+	+	+	-	+	-	+	+	+	+	-	+	+	+	-
4	17	8.3	+	-	+	+	+	-	+	-	-	-	+	+	+	+	+	+
4	1		+	-	+	+	+	-	+	-	-	+	-	+	+	+	+	+
6	8	4.6	+	+	+	+	+	+	+	+	+	-	+	+	+	+	+	+
6	2		+	+	+	+	+	+	+	+	+	+	-	+	+	+	+	+
7	3	3.6	+	+	+	+	+	-	+	+	+	-	+	-	+	+	+	+
7	4		+	+	+	+	+	+	+	+	+	-	+	-	+	+	+	+
7	1		+	+	+	+	+	+	+	+	+	+	-	-	+	+	+	+
12	8	3.6	+	+	+	+	+	-	+	+	-	-	+	+	+	+	+	+

In particular, genotype 1, observed in 62 samples with a frequency of 28.5%, closely resembles that observed in the other Caucasian, Palestinian and Thai populations, but not in the Indian and Aborigine populations, which have a frequency of 5.6% and 1.7%, respectively [5,6,8,15,16]. In this genotype the expression of *KIR2DS4 *is related to the homozygous allele *003 in 39 out of 62 and in 20 out of 62 samples in heterozygous combinations with allele *001/*002.

Genotype 2 was present in 28 people with a frequency of 12.9%; it includes the inhibitory gene *KIR2DL2 *and has a frequency higher than that observed in other Caucasian and non-Caucasian populations or in the Palestinian and Thai populations which have a similar frequency [6,8,15]. In this case KIR2DS4*003 is in heterozygous and homozygous combinations in 11 and 16 out of 28 individuals, respectively.

Genotype 3 with a frequency of 9.2% (20 samples) always includes gene *KIR2DL2 *and has a frequency that is higher than the Australian Caucasian, Greek and German populations, while it has a similar percentage to that observed in another German study and in a Palestinian population study [5,6,19]. Genotypes included *KIR2DL5 *which is only positive in the variant *KIR2DL5B*, while *KIR2DS4 *is positive for the homozygous allele *003 in 16 samples.

Genotype 4, observed in 18 people with a frequency of 8.3%, is lower than that observed in Australian Caucasian and Irish populations but similar to that observed in other Caucasian and non-Caucasian studies [6,8,14]. In this genotype *KIR2DL5 *is always positive in the variant *KIR2DL5A *while *KIR2DS4 *is positive in homozygous combination for the allele *003 in 80% of this genotype.

Genotype 6 includes all inhibitory and non-inhibitory genes and is present in 10 cases (4.6%) somewhat similar to the position observed in the Caucasian and non-Caucasian populations but not in the Australian Aborigine [6]. In this genotype the two variants of *KIR2DL5 *are equally expressed while KIR2DS4*003 is present in 8 out of 10 cases.

Genotype 7, which includes all inhibitory and non-inhibitory genes except *KIR2DS5*, has a frequency of 3.6% which is lower than has been observed in other Caucasian populations, similar to the finding in Palestinian and Thai studies and much lower than in the Australian Aborigine population [6-8,15].*KIR2DL5A *is positive in all samples and *KIR2DL5B *in about half. Allele KIR 2DS4*003 is observed in 7 out of 8 samples.

Genotype 12 has a percentage of 3.6%, similar to that observed in other Caucasian and non-Caucasian populations except in Australian Caucasians where it is higher [6]. In these 8 samples we only have positivity in genes *KIR2DL5A *and KIR2DS4*003.

Genotypes 5 and 9, which are characterized by positivity for *KIR2DL1 *and *KIR2DL2 *and negativity for *KIR2DL3*, have a frequency of 2.3% and 1.3% respectively.

The A putative homozygote haplotypes in the Italian population are the most prevalent (57%) and the distribution of genotypes in terms of combination of haplotypes proves to be AA 28.5%, AB 69.5% and BB about 2% [10].

Pseudogene typing shows a close correlation between *KIR2DP1 *and haplotype A while it is absent in haplotype B. Haplotype A is also characterized by the presence in all samples of allele KIR3DP1*003, while genotype B in our four samples only shows positivity for KIR3DP1*001/002 [21].

## Discussion

In this work we evaluated the percentage of the population positive for each KIR gene as well as the KIR genotype frequency in a representative ethnic Italian group of 217 randomly selected unrelated healthy individuals from 22 immunogenetics laboratories, located in northern, central and southern regions of Italy.

All the participating laboratories studied the two shared DNA samples and the results showed typing consensus. Each KIR gene was detected by a single primer mix and no ambiguities were observed. Management of the new kit was acceptable by all laboratories.

The percentage of the population positive for a KIR gene was compared to other Caucasian and non-Caucasian populations [5-8,14-20]. While the differences in terms of low percentages observed in isolated genes could be connected with the primers that we used in typing, since these may have recognized alleles that were not evaluated in early published studies, the opposite should not apply: where older methods developed more positivity than ours (as with *KIR2DL2 *in the Australian Aborigine and North Indian populations), our results must be taken as significant.

The three populations that show most markedly different patterns from ours are the Australian Aborigine, Chinese Han and Japanese, in particular for inhibitor genes *KIR2DL2 *and non inhibitor *KIR2DS1*, *KIR2DS2*, *KIR2DS3*, *KIR3DS1*, which have a significant difference from our Italian study, as indeed from other Caucasian population evaluations [6,17,18].

For the Chinese Han population study the same primers were used as in our own and hence form a valid point of comparison. In particular, inhibitor genes such as *KIR2DL2 *and *KIR2DL5B *were found in an extremely low percentage, as was the non inhibitor gene *KIR2DS3*. *KIR2DS4 *alleles are especially interesting since the Chinese pattern is exactly the inverse of the Italian [17].

*KIR2DL1 *and *KIR2DL3 *are observed in high positive linkage disequilibrium as detected in other Caucasian and non-Caucasian populations and this datum further suggests a segregation of KIR region haplotypes into two groups distinguished by *KIR2DL1/KIR2DL3 *and *KIR2DL2*, although the latter is claimed to have arisen as a recombination event between *KIR2DL1 *and *KIR2DL3 *[22].

A negative linkage disequilibrium was identified between KIR2DS4*001/002 and KIR2DS4*003, suggesting their alleles may not be identical, as already confirmed in other Caucasian and non-Caucasian populations [23].

The KIR repertoire is substantially influenced by the polymorphic and polygenic nature of the KIR gene. However, using primers to identify the two variants of *KIR2DL5 *(2DL5A, 2DL5B) and the allele KIR2DS4*003, each genotype described by Uhrberg might be assigned to a haplotype with more than one combination [4,23,24].

In this study we observed that genotype 1is the most representative, as in other Caucasian populations, and our data are in agreement with other studies on Caucasian populations concerning the predominance of the haplotype A group over the haplotype B group [5]. This means that 28.5% of the Italian population have *KIR2DS4 *as the only activating gene. Bearing in mind the high percentage of individuals who have only the deleted form of *KIR2DS4*, this would lead to at least 18% of this population not having a functional activating receptor, provided that the assumption of the deleted form of *KIR2DS4 *not being expressed is correct [23].

Genotype 1 is also the most frequent in the Chinese Han non-Caucasian population but the percentage is higher than observed in our study (58.7% vs 28.5%). In this genotype the distribution of the *KIR2DS4 *alleles has a completely contrary pattern to what we observed in our population, suggesting a different positivity of the alleles but also a different haplotype recombination in the populations [17]. The particular distribution of KIR genes could be due to a selection pressure, which would favour a particular combination of KIR genes, and this phenomenon could be explained by the linkage disequilibium observed.

The actual number of genotypes in the Italian study population is likely to be much lower higher than we surmised because the medium level of resolution was only studied for *KIR2DL5 *and *KIR2DS4*. Typings for the two groups, alleles *KIR2DL5 *and *KIR2DS4*, already show an increase in the variety of KIR gene profiles. Moreover the distribution of the patterns described should be revised because the analysis in which there was no or low discrimination of alleles will have overestimated the similarities and underestimated the differences between populations [25,26].

Combinations of particular HLA-KIR genotypes have also been linked with susceptibility to autoimmune diseases such as psoriatic arthritis and type I diabetes, just as several genetic studies have revealed an influence of HLA-KIR gene interactions on disease outcome.

Interactions between HLA-Bw4 and *KIR3DS1 *gene have been observed in cases of delayed progression to AIDS in HIV patients, while the homozygosity of both HLA-C1 and *KIR2DL3 *is associated with resolution of hepatitis C virus infection. Understanding the basis for these observed genetic associations is complicated by the extensive polymorphism found among KIR haplotypes, which differ not only in nucleotide sequence but also in gene content [27].

In having established the percentage of the population positive for a KIR gene and the distribution of KIR genotyping, the present study may serve as a reference for other studies of genetic associations between KIR genes and specific diseases.

Recently several reports have described a role for KIR genes in bone marrow transplantation. At present the approach to identification of NK alloreactive donors in haploidentical bone marrow transplants has been based on KIR ligand incompatibility [11]. However this incompatibility has also been shown in HLA-identical sibling transplantation [28]. Therefore the availability of a simple, reproducible molecular biology test such as we have used in this study to type the KIR genes could become part of donor evaluation and selection, since it directly identifies the presence or absence of a given KIR gene in the donor. One important example of this is the lack of the *KIR3DL1 *gene for HLA-Bw4 inhibitory receptors, which immediately excludes about 4% of potential donors.

In broad conclusion, our study shows that the Italian population possesses the general KIR gene features reported in other Caucasian and non-Caucasian populations, but the discrepancies in the case of certain genes confirm the polymorphic and polygenic nature of the KIR genes in the population.

## Abbreviations

KIR: Killer cell immunoglobulin-like receptors

HLA: Human Leukocyte Antigen

PCR-SSP: polymerase chain reaction sequence specific primer

## Authors' contributions

AB and MT organized the collaborative study, carried out the molecular genetic study and drafted the manuscript; MCC and MM carried out the molecular genetic study and helped to draft the manuscript; CC, AC, EC, ED, SF, AMI, CL, AM, MM, LM, IM, LM, VM, SN, GO, DP, GR, CT and SV carried out the molecular genetic study, while MA and RC helped to draft the manuscript.  
